# A high coverage reference transcriptome assembly of pea (Pisum sativum L.) mycorrhizal roots

**DOI:** 10.18699/VJ20.625

**Published:** 2020-07

**Authors:** A.M. Afonin, I.V. Leppyanen, O.A. Kulaeva, O.Y. Shtark, I.A. Tikhonovich, E.A. Dolgikh, V.A. Zhukov

**Affiliations:** All-Russia Research Institute for Agricultural Microbiology, Pushkin, St. Petersburg, Russia; All-Russia Research Institute for Agricultural Microbiology, Pushkin, St. Petersburg, Russia; All-Russia Research Institute for Agricultural Microbiology, Pushkin, St. Petersburg, Russia; All-Russia Research Institute for Agricultural Microbiology, Pushkin, St. Petersburg, Russia; All-Russia Research Institute for Agricultural Microbiology, Pushkin, St. Petersburg, Russia Faculty of Biology, St. Petersburg State University, St. Petersburg, Russia; All-Russia Research Institute for Agricultural Microbiology, Pushkin, St. Petersburg, Russia; All-Russia Research Institute for Agricultural Microbiology, Pushkin, St. Petersburg, Russia

**Keywords:** RNAseq, transcriptomics, arbuscular mycorrhiza, garden pea, РНК-секвенирование, транскриптомика, арбускулярная микориза, горох посевной

## Abstract

Arbuscular mycorrhiza (AM) is an ancient mutualistic symbiosis formed by 80–90 % of land plant species with the obligatorily biotrophic fungi that belong to the phylum Glomeromycota. This symbiosis is mutually beneficial, as AM fungi feed on plant photosynthesis products, in turn improving the efficiency of nutrient uptake from the environment. The garden pea (Pisum sativum L.), a widely cultivated crop and an important model for genetics, is capable of forming triple symbiotic systems consisting of the plant, AM fungi and nodule bacteria. As transcriptomic and proteomic approaches are being implemented for studying the mutualistic symbioses of pea, a need for a reference transcriptome of genes expressed under these specific conditions for increasing the resolution and the accuracy of other methods arose. Numerous transcriptome assemblies constructed for pea did not include mycorrhizal roots, hence the aim of the study to construct a reference transcriptome assembly of pea mycorrhizal roots. The combined transcriptome of mycorrhizal roots of Pisum sativum cv. Frisson inoculated with Rhizophagus irregularis BEG144 was investigated, and for both the organisms independent transcriptomes were assembled (coverage 177x for pea and 45x for fungus). Genes specific to mycorrhizal roots were found in the assembly, their expression patterns were examined with qPCR on two pea cultivars, Frisson and Finale. The gene expression depended on the inoculation stage and on the pea cultivar. The investigated genes may serve as markers for early stages of inoculation in genetically diverse pea cultivars.

## Abstract (ru)

Арбускулярная микориза (AM) – это древний мутуалистический симбиоз, который образуют 80– 90 % видов наземных растений с облигатно биотрофными грибами, принадлежащими к филе Glomeromycota. Этот симбиоз является взаимовыгодным, поскольку грибы AM питаются продуктами фотосинтеза растений, в свою очередь повышая эффективность поглощения питательных веществ растением из окружающей среды. Горох (Pisum sativum L.), широко распространенная сельскохозяйственная культура и важный модельный объект генетики, способен образовывать тройные симбиотические системы, состоящие из растения, грибов AM и клубеньковых бактерий. По мере распространения транскриптомных и протеомных подходов в изучении мутуалистических симбиозов гороха, для повышения разрешающей способности и точности других методов возникла необходимость в референсном транскриптоме, т.е. в знании последовательностей генов, экспрессирующихся в различных экспериментальных условиях. Многочисленные транскриптомные сборки, сконструированные для гороха, не включали микоризованные корни, поэтому целью данного исследования было создание референсной транскриптомной сборки микоризованных корней. Было проведено глубокое РНК-секвенирование транскриптома микоризованных корней Pisum sativum сорта Frisson, инокулированных Rhizophagus irregularis BEG144, и для каждого из организмов получены независимые транскриптомные сборки (покрытие 177х для транскриптома гороха и 45x для транскриптома гриба). Качество сборки транскриптома гороха оценено путем сравнения с уже имеющимися сборками транскриптомов других тканей. Для дополнительной оценки качества сборки, у двух сортов гороха (Frisson и Finale) с помощью qPCR проведен анализ экспрессии генов, специфичных для микоризованных корней, последовательности которых были найдены в созданной сборке. Исследованные гены могут служить маркерами ранних стадий развития арбускулярной микоризы у генетически разнообразных сортов гороха.

## Introduction

Plants are able to establish mutualistic association with the arbuscular mycorrhizal (AM) fungi that improve the efficiency of nutrient uptake from the environment. About 80–90 % of all land plant species may form mutually beneficial symbiosis with the obligatorily biotrophic AM fungi that belong to the phylum Glomeromycota (Kaschuk, 2009; Alizadeh, 2011; Tisserant et al., 2012; Gutjahr, Parniske, 2013; Manck-Götzenberger, Requena, 2016). The AM facilitates plant nutrition and increases plant tolerance to biotic and abiotic stresses, when AM fungi feed on photosynthesis products and utilize a considerable proportion of the assimilated carbon (Siddiqui et al., 2008; Solaiman et al., 2014).

The garden pea (Pisum sativum L.) is a widely cultivated crop plant and the first model object of genetics. Similarly to other legume plants belonging to the Fabaceae family, it is capable of forming triple symbiotic systems consisting of plant, AM fungi and nodule bacteria (Tikhonovich et al., 2015). The formation of symbioses increases yield and plant fitness in general (Jacobi et al., 1999; Borisov et al., 2004; Shtark et al., 2006; Zhukov et al., 2019), although the effect of inoculation is often dependent on experimental conditions and plant genotype (Shtark et al., 2006; Zhukov et al., 2017; Mamontova et al., 2019). During the last decade, transcriptomic and proteomic approaches had been implemented for studying the mutualistic symbioses of pea, which implied the need for a reference genomic or transcriptomic sequences required for proper annotation of transcripts/proteins under analysis. Although numerous transcriptome assemblies were constructed (Franssen et al., 2011; Alves-Carvalho et al., 2015; Sudheesh et al., 2015; Zhukov et al., 2015; Kerr et al., 2017), no samples containing mycorrhizal roots had been analysed. Moreover, the only available genomic sequence (Kreplak et al., 2019) is far from ideal and lacks sequences of many important genes (for example, short peptides of NCR and defensin families).

Thus, the present work aimed at obtaining the reference transcriptome assembly of pea mycorrhizal roots using RNAseq. In order to validate the constructed assembly, which is destined for further analysis of the mycorrhization in pea with use of transcriptomics and/or proteomics, we designed the set of primers for qPCR expression analysis and successfully quantitated the expression level of 10 AM-specific genes in mycorrhizal roots of two pea cultivars.

## Materials and methods

**Plants and microorganisms used.** The wild-type Pisum sativum L. cultivars Frisson (= JI2491 (Duc, Messager, 1989)) and Finale (= JI2678 (Engvild, 1987)) were used in this study. The fungal isolate Rhizophagus irregularis BEG144 was provided by the International Bank for the Glomeromycota (Dijon, France) as a substrate-root based inoculum for leek (Allium porrum L.) pot cultures. It was used to obtain nurse pots of chives (Allium schoenoprasum L.) for the R. irregularis-inoculated pea plants (according to (Shtark et al., 2016))

**Plant growth conditions and root sampling.** In order to provide an efficient inoculation of P.sativum plants, nurse pots with established mycorrhiza were used. These were 300-ml ceramic flower pots filled with opoka-rock mineral substrate, which is silica rich marl (Krasnodar, Russia), supplemented with 1 g .L^–1^ calcium orthophosphate. Prior to nurse pots preparation, pots with substrate were sterilized by autoclaving for 60 min at 134 °C and 0.22 MPa. Procedures for the chivebased nurse pots are described by (Demchenko et al., 2004; Shtark et al., 2016).

Seeds of P. sativum were surface-sterilised for 10 minutes with 98 % sulphuric acid, rinsed with sterile deionised water five times, and then germinated for 3 days at 27 °C in the dark on sterile vermiculite in Petri dishes with 30 ml of water added to each one. Three P. sativum seedlings of similar size were planted into each nurse pot around a mycorrhizal chive plant. Plants were grown in a growth chamber (model VB 1514, Vötsch, Germany) under the following conditions: day/night, 16/8 h; temperature, 24/22 °C; relative humidity, 75; irradiation 10000 lux and were supplemented once a week with 1/2-x Hoagland’s solution (Hoagland, Arnon, 1950) without phosphate (50 mL per pot), and watered as needed.

After several days of co-cultivation with chives in nurse pots P.sativum root samples were collected for transcriptome analyses and RT-qPCR (see relevant sections) and immediately frozen in liquid nitrogen and stored at –80 °C. Frozen root samples were ground in liquid nitrogen using pestle and mortar. Before collecting plant material for those analyses, several lateral roots (15 cm length) from each pea root system were randomly selected and frozen at –20 °C and then subjected to analysis of AM development as described by Shtark and colleagues (Shtark et al., 2016). The parameters of root colonization of these pea plants estimated according to (Trouvelot et al., 1986) were: M % (intensity of internal colonization of the root system), and a % (arbuscule abundance in mycorrhizal root fragments)

For transcriptome sequencing root samples were collected after 25 days of co-cultivation with chives. Three plants of cv. Frisson with M % = 68.6±2.6, and a % = 50.9±1.2 (this level of root colonization is common with this genotype (Morandi et al., 2000; Grunwald et al., 2004)), were chosen for the transcriptome analysis. The whole root systems were cut off directly below the cotyledons and frozen in liquid nitrogen in 50 mL Falcon tubes.

For RT-qPCR assays root samples were collected after 7, 14 or 28 days of co-cultivation with chives. Root samples of uninoculated plants grown under the same conditions during the same time periods were used as a control. Lateral roots were used, in which tips 1 cm long were removed. The collected samples were frozen in 2 mL Eppendorf™ tubes. The AM development was analysed at two time points (after 14 and 28 days of co-cultivation with chives). The parameters of cv. Finale root colonization were: M % = 17.5±2.3, and 35.0±4.7, respectively; a % = 29.4±5.7, and 36.5±5.7, respectively. This level of colonization is common with cv. Finale (Shtark et al., 2016; Leppyanen et al., 2018). The parameters of cv. Frisson root colonization were: M % = 44.6±4.7, and 68.1±5.4, respectively; a % = 62.6±3.5, and 65.3±4.4, respectively. This is also consistent with previous investigations; in particular it was shown that internal AM colonization and arbuscule development reaches higher values in cv. Frisson than in cv. Finale (Morandi et al., 2000; Grunwald et al., 2004)

**Transcriptome sequencing.** The RNA extraction, library construction and the sequencing on an Illumina 2500 HiSeq platform was carried out by GenXPro GmbH (Frankfurt-amMain, Germany)

**Read preparation.** In order to remove possible human and bacterial contaminants from the raw data we used the method, devised by dr. Brian Bushnell and described in removeHuman tool from the BBTools suite (Bushnell, 2014). Additionally, reads belonging to bacteria and viruses were discarded using the databases provided by the author of the package. In order to separate fungal reads from those of pea, we first prepared the genome assembly of Rhizophagus irregularis strain DAOM197198 from (Tisserant et al., 2013) as described by the author of the bbmap package. The areas of genome, containing multiple tandem short k-mers, as well as windows of low entropy sequences, which were calculated using pentamer frequencies, were masked using the bbmask.sh script. In order to exclude the sequences, common between the R. irregularis genome and plant genomes, all the available non-draft plant genomes from the Phytozome V12 (Goodstein et al., 2012) were masked for repetitive sequences, and then used to exclude all the non-specific parts of the DAOM197198 genome as described for the bbmask.sh script. The resulting masked assembly was used for read mapping using the bbmap.sh script. All the mapped reads were considered to belong to the fungus, all the non-mapped reads were considered plant reads. The transcriptome completeness and assembly quality were assessed using BUSCO algorithm (Simão et al., 2015). Blast search was used for the comparison of the various transcriptomes.

**Transcriptome assembly.** The reads belonging to Pisum were then assembled using Trinity assembler (v2.6.6) (Grabherr et al., 2011) with default parameters, rnaSPAdes (v3.11.1) (Bankevich et al., 2012). Corset/Lace pipeline was used to assemble the SuperTranscriptome (Davidson et al., 2017).

The transcriptome shotgun assemblies (TSAs) from the following bioprojects were downloaded from the NCBI for comparison to current assembly: PRJNA277074 – cv. Kaspa (Sudheesh et al., 2015), PRJNA277076 – cv. Parafield (Sudheesh et al., 2015), PRJNA308776 – cv. Torsdag (Kerr et al., 2017). The assembly of the nodule transcriptome of cv. SGE (accession GDTM00000000.1) and the assembly of roots of cv. SGE (accession GDTL00000000.1) were also downloaded from the NCBI (Zhukov et al., 2015). Additionally, the assemblies from cv. Caméor (Alves-Carvalho et al., 2015) and cv. Little Marvel (Franssen et al., 2011) were downloaded from supplementary files for the respective articles. The CDS sequences from the M. truncatula v4.0v2 genome were used for benchmarking the assembled transcriptomes (Young et al., 2011).

The following accessions were downloaded from the NCBI in order to identify the R. irregularis strain closest to BEG144: GCA_000439145.3, GCA_000597565.1, GCA_000597585.1, GCA_000597605.1, GCA_000597625.1, GCA_000597645.1, GCA_000597665.1, GCA_000597685.1, GCA_001593125.1, GCA_001593145.1, GCA_001593155.1, GCA_001593205.1, GCA_002897155.1, GCA_003833045.1, GCA_003833115.1. The reads binned as belonging to the fungi were assembled denovo using the Trinity assembler. Cufflinks (v2.2.1) (Trapnell et al., 2010) was used to build a genome-guided assembly

**Annotation.** CDS discovery for both the organisms was performed using the transdecoder (v5.2.0) algorithm (https://github.com/TransDecoder/TransDecoder/). Both the hmm and the blast homology search options were used, according to the manual. The annotation was performed using the EggNOG 5.0 database using the eggNOG-mapper (v2.0.1) (Huerta-Cepas et al., 2016)

In order to determine the best overall assembly, the three transcriptomes were evaluated using the BUSCO tool (Simão et al., 2015)

**RT-qPCR.** Total RNA was isolated using the TriZol reagent (ThermoScientific, USA). cDNA synthesis was performed with reverse transcriptase (ThermoScientific, USA) and oligodT primers (Evrogen, Russia; www.evrogen.com). RT-qPCR was performed on the CFX-96 C1000 thermocycler (BioRad Laboratories, USA) with the double-stranded DNA dye SYBR Green (Bio-Rad Laboratories, USA). The data was analysed using the 2-ΔΔCt method (Livak, Schmittgen, 2001). PCR amplification was confirmed with the dissociation curve method (55 to 95 °C). mRNA levels were normalised in relation to the ubiquitin and actin reference genes. Three biological replicates were analysed. The primers were constructed using the Vector NTI suite (Lu, 2004) (Table 1). The expression levels of the genes of interest (GOI) relative to the reference genes Ubiquitin and Actin were calculated for each cDNA sample using the CFX Manager™ software version 2.1 (BioRad Laboratories, USA). The expression levels of GOI were calculated as ratio of treated samples to control samples. Statistical analysis was conducted by SIGMAPLOT13 (Systat Software, Inc., San Jose California, USA).

**Table 1.**
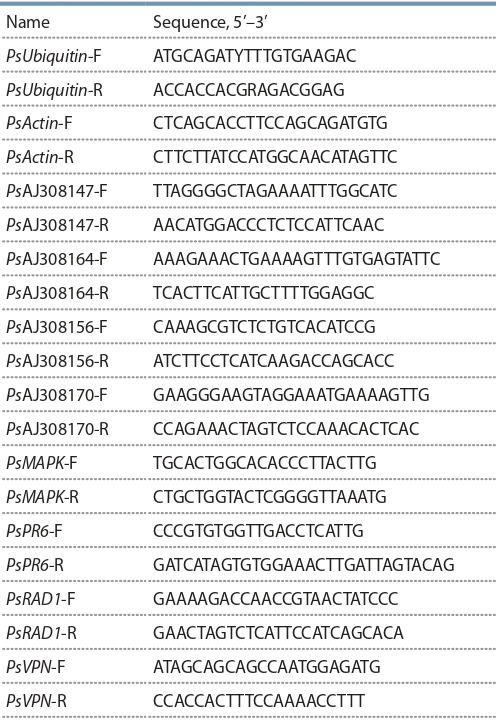
Constructed primers used for qPCR Note: Melting point (Тm) for every primer was 54 °C.

## Results and discussion

In order to obtain a reference transcriptome of the mycorrhizal roots of P. sativum, the RNA from this tissue was sequenced on an Illumina 2500 sequencing platform

A total of 120 mln pairs of reads with average length of 150 bp were obtained. After quality trimming 119 mln reads remained. Since we knew, that R. irregularis transcripts are present in the tissues, we decided to remove all possible contaminants (as described in the Materials and methods section). Additionally, we masked low complexity regions and repetitive elements from R. irregularis genome. Of the 119 mln remaining reads 6.7 mln (approximately 5 %) of the paired reads were mapped onto the masked Rhizophagus genome.

The resulting reads belonging to P. sativum were then assembled using two assemblers: Trinity with default parameters and rnaSPAdes with default parameters. Afterwards, with use of Corset/Lace pipeline, a SuperTranscripts assembly was constructed using these two assemblies comprising 94360 supertranscripts (Table 2).

**Table 2.**
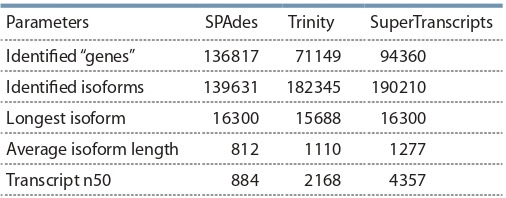
Comparison of transcriptome assemblies’ parameters Note: The estimated coverage for the transcriptome was 177x

## Transcriptome evaluation

In order to determine the best overall assembly, the three transcriptomes were evaluated using the BUSCO tool. Results of the analysis are presented in the Table 3.

**Table 3.**
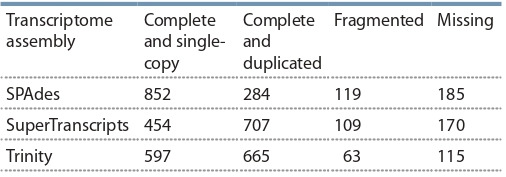
The comparison of the transcriptome assemblies using the BUSCO tool

As BUSCO tends to favor the number of transcripts, the Trinity assembly scored higher than the SuperTranscripts assembly, despite the latter containing the former. The other comparisons, such as N50 and the duplication numbers speak in favor of the SuperTranscripts assembly

The resulting SuperTranscripts assembly, as well as the other two assemblies, the table of relation between the assemblies and the full results of are available at the (https://cloud.arriam.ru/s/RKD67CZak58BzzN)

Since the SuperTranscript assembly contains the two other assemblies, it was used to compare to all available pea transcriptome assemblies. The comparison was performed using the BLASTN algorithm (e-value < 10^–10^, identity > 90 %, query coverage > 90 %), the results are presented in the Table 4.

**Table 4.**
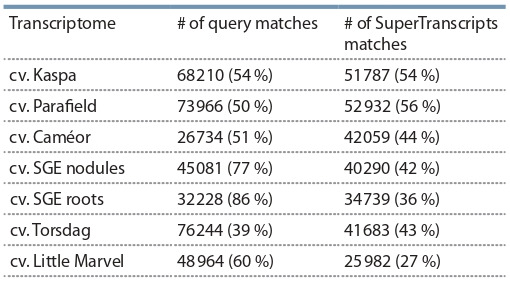
The results of comparison of the SuperTranscripts assembly to the other available transcriptome assemblies

The low numbers of query matches in the case of the cv. Caméor is most probably due to the fact, that the transcriptome in this study represents a single, rather than multiple, plant tissues.

All the available pea transcriptomes were compared to the latest Medicago truncatula Gaertn. transcriptome in order to determine the fullness of the transcriptomes and the number of novel transcripts (Table 5). Our assembly had an almost identical number of transcripts, homologous to those of M. truncatula as the more diverse transcriptomes of cv. Kaspa, cv. Parafield, and cv. Caméor, and also showed a large number of previously undiscovered transcripts

**Table 5.**
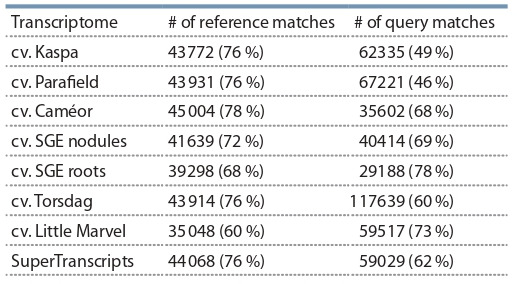
The results of comparison of the pea transcriptome assemblies (query) to the M. truncatula transcriptome (reference) Note: e-value < e^–10^, query coverage > 80 %, identity > 60 %.

## Determining the specificity of inoculation

In order to check for possible rhizobial contamination and formation of nodules possibly missed during sample preparation we decided to search for nodule specific proteins. Since the expression counts for a single RNAseq analysis cannot be considered statistically significant, we chose instead to search for contigs, encoding proteins, specific to nodules – NCR peptides (Zorin et al., 2019). Using the approach from that article, we found only 28 genes of the NCR gene family in the newly assembled transcriptome, compared to 40 in the cv. SGE root tips and 425 in the cv. SGE nodules. Therefore, it is reasonable to assume that the plants were not accidentally infected with rhizobia, and thus are representative of a fungal monoinoculation.

**Novel transcripts of Rhizophagus irregularis**

Since for now there is no published genome available for the strain BEG144 used in this study, we decided to determine the closest related strain in the NCBI database. To do this, we downloaded all the available R. irregularis genomes and mapped the filtered transcripts using bbmap.sh to the genomes. The best overall mapping was to the strainA1 of R. irregularis GCA_001593125.1 (registered bioproject url: https://www.ncbi.nlm.nih.gov/bioproject/PRJNA299202/). This genome was used for a genome-guided Cufflinks assembly. The mapping results are presented in the Table 6.

**Table 6.**
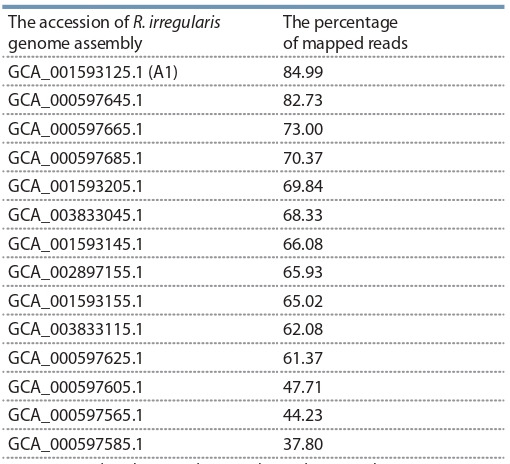
The results of mapping of fungal reads onto the transcriptome assemblies of R. irregularis from the NCBI Note: BBmap.sh tool was used to map the reads against the genomes.

Genome guided Trinity assembly did not yield any assembled contigs, possibly due to the lower coverage of the fungal genome, so a de-novo assembly of the fungal transcriptome was performed. The results of the assemblies are present in the Table 7.

**Table 7.**
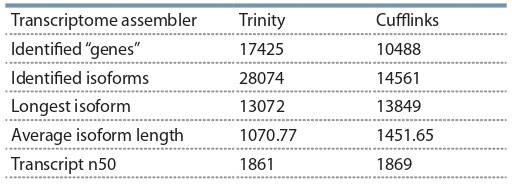
Comparison of R. irregularis transcriptome assemblies

The likely protein-coding genes from the assemblies were identified by transdecoder (see Materials and methods section). The Cufflinks assembly contained 12036 (77.8 %) protein-coding genes, whereas the Trinity assembly contained 17909 (63.7 %). After comparing the assemblies with the blast algorithm we discovered that 8301 (68 %) of Cufflinks CDS-containing transcripts corresponded to 12036 (60.1 %) of CDS-containing transcripts of Trinity. The rest of the transcripts, unique to the Trinity assembly may represent the parts of genome different between the strain BEG144 and the strain used as the reference. The low coverage and the unavailability of the genome of the strain BEG144 make it harder to distinguish the real transcripts from the chimeric. The results, however, show the usability of proposed methods to assemble the transcriptomes of mixed samples, even in the absence of high-quality reference genomes.

## RT-qPCR of AM-specific genes

In order to study the inoculation effects in detail and to monitor the AM development during the experiments, the set of marker genes with AM-specific expression pattern was required. In previous work by Grunwald et al. (2004), a set of 25 genes which were upregulated more than in 2.5 times in response to inoculation with AM fungus in roots of pea cultivar Finale was identified using suppressive subtractive hybridization. We selected eight of them for the present study and supplemented this list with the well-characterized mycorrhiza-related genes encoding the transcription factor RAD1 and the VAPYRIN protein for analysis (Pumplin et al., 2010; Murray et al., 2011; Park et al., 2015). In order to find the sequences of 10 AMspecific genes of cv. Frisson, a BLASTN search of corresponding genes was performed against the newly created SuperTranscripts transcriptome assembly. The accessions are presented in Table 8. We compared the RT-PCR expression patterns for chosen genes in cv. Finale as well as cv. Frisson pea plants (Grunwald et al., 2004). The used primers are presented in the Table 1.

**Table 8.**
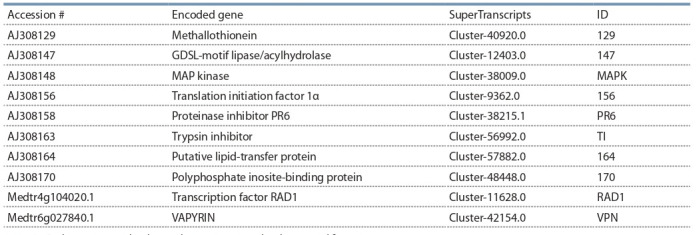
Genes chosen according to (Grunwald et al., 2004) and their corresponding accessions in the SuperTranscripts assembly

**Fig. 1.**
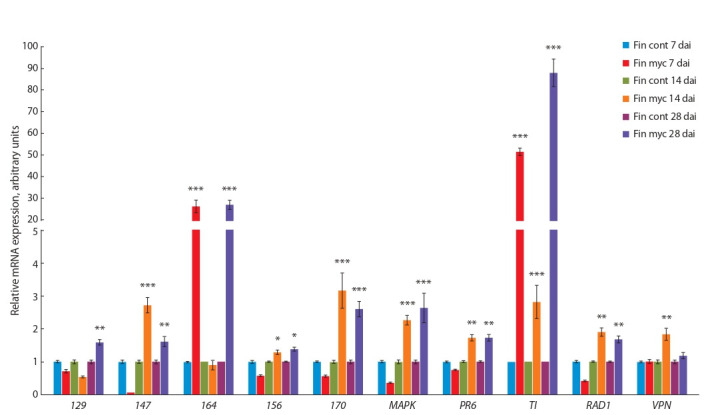
Transcript levels of genes encoding markers of AM development in pea roots of cv. Finale infected with R. irregularis after 7, 14 and 28 days Here and in Figure 2: uninoculated plant roots were used as control (cont); the treatment was mycorrhization (myc). The relative expression was normalized against the constitutively expressed ubiquitin and actin genes. Values are means±SEM of three technical repeats. The graphs show the results of one biological replication, representative of three biological independent experiments, the asterisks indicate the significant differences between control and treatment as analysed by Student’s t-test (***p < 0.001, **p < 0.01, *p < 0.05).

**Fig. 2.**
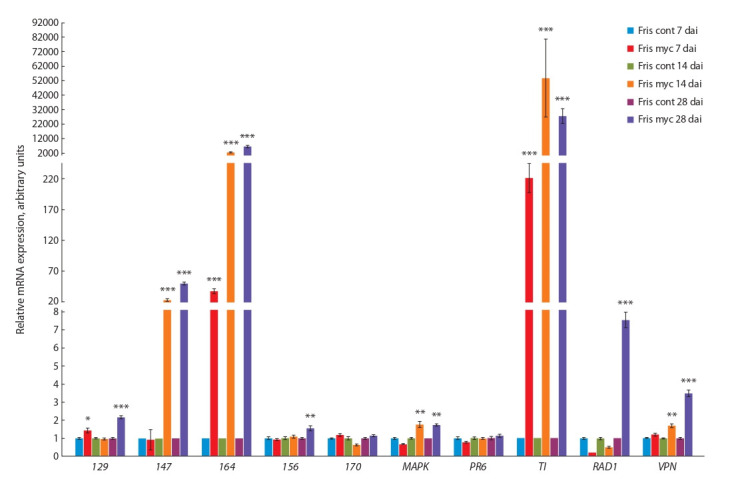
Transcript levels of genes encoding markers of AM development in pea roots of cv. Frisson infected with R. irregularis after 7, 14, and 28 days

This allowed the identification of predicted full-length sequences for genes of interest. The expression of 10 genes was investigated by RT-PCR using RNA from control and inoculated roots of cv. Finale and cv. Frisson on several days after inoculation (dai) (Figure 1, 2). The cv. Finale was chosen to compare the results with previously obtained data for this genotype, while cv. Frisson was used for analysis, because the newly created transcriptome was assembled for this cultivar. Indeed up-regulation of a number of genes was confirmed by RT-PCR using RNA from controls and mycorrhiza-inoculated pea roots of cv. Frisson and cv. Finale. The expression of RAD1 and VAPYRIN genes was mainly enhanced at stages of symbiosis development related to AM fungal colonization of root cells and arbuscules formation (14–28 dai). The similar results were previously obtained for model legume plant M. truncatula (Pumplin et al., 2010; Murray et al., 2011; Park et al., 2015)

The highest levels of expression were found for four genes encoding GDSL-motif lipase/acylhydrolase (147 ), MAP kinase (MAPK ), trypsin inhibitor (TI ) and putative lipid-transfer protein (164 ) in both genotypes (see Figures 1, 2). It is in a good agreement with previous results (Grunwald et al., 2004). It is interesting to note that activation of TI and 164 was connected with early stages of AM symbiosis development such as 7 dai. These markers may be helpful to estimate the inoculation effect, because usually visible signs of inoculation seem to be connected with later stages of symbiosis between pea plants and AM fungi like 14 dai. At the same time some cultivar-specific mycorrhiza-related pattern of expression was found in our experiments. It was shown for gene 170 with high level of expression in cv. Finale, but not in cv. Frisson (see Figure 1, 2). Moderate level of expression was shown for the stress-related genes like 129 and PR6 in our experiments that may be due to differences in experimental conditions as compared to previous study (Grunwald et al., 2004).

## Conclusion

Studies on model legume plants M. truncatula and Lotus japonicus (Regel.) K. Larsen resulted in the description of the molecular mechanisms underlying the formation and functioning of the symbioses (Gutjahr, Parniske, 2013; Gobbato, 2015; Pimprikar, Gutjahr, 2018). However, for the agriculturally important legume plants, the information regarding the molecular bases of AM and root nodule symbiosis (RNS) is still limited. As for the garden pea, about 40 regulatory symbiotic genes are known (Zhukov et al., 2016), of which about a half are attributed to the ‘common’ symbiotic genes needed for both AM and RNS (Borisov et al., 2007). The recent development of transcriptome sequencing technologies has made it possible to analyze the molecular machinery of symbiotic nodules and, in particular, to pinpoint the single nucleotide deletion mutation in important symbiotic gene Sym33 (IPD3) (Zhernakov et al., 2019). Our first assembled transcriptome of mycorrhizal pea roots will help in characterization of the key plant genes involved in the regulation of mycorrhizal symbiosis and, therefore, will be a basis for the future advances in our understanding of plant-microbe interactions. The AM-specific genes that we selected on the base of this reference transcriptome may also serve as markers of successful colonisation in inoculation experiments, which is important for analysing the early stages of mycorrhization, when intraradical mycelium is almost invisible.

## Conflict of interest

The authors declare no conflict of interest.
